# Cement-augmented dorsal instrumentation of the spine as a safe adjunct to the multimodal management of metastatic pheochromocytoma: a case report

**DOI:** 10.1186/1754-9493-6-1

**Published:** 2012-01-05

**Authors:** Daniel Rittirsch, Edouard Battegay, Lukas U Zimmerli, Werner Baulig, Donat R Spahn, Christian Ossendorf, Guido A Wanner, Hans-Peter Simmen, Clément ML Werner

**Affiliations:** 1 Division of Trauma Surgery, University Hospital Zurich, Switzerland; 2 Division of Internal Medicine, University Hospital Zurich, Switzerland; 3 Institute of Anesthesiology, University Hospital Zurich, Switzerland

**Keywords:** malignant pheochromocytoma, thoracic spine, adrenergic blockade, dorsal instrumentation, cement augmentation

## Abstract

Malignant pheochromocytoma is a neuroendocrine tumor that originates from chromaffin tissue. Although osseous metastases are common, metastatic dissemination to the spine rarely occurs.

Five years after primary diagnosis of extra-adrenal, abdominal pheochromocytoma and laparoscopic extirpation, a 53-year old patient presented with recurrence of pheochromocytoma involving the spine, the pelvis, both proximal femora and the right humerus. Magnetic resonance imaging and computed tomography revealed osteolytic lesions of numerous vertebrae (T1, T5, T10, and T12). In the case of T10, total destruction of the vertebral body with involvement of the rear edge resulted in the risk of vertebral collapse and subsequent spinal stenosis. Thus, dorsal instrumentation (T8-T12) and cement augmentation of T12 was performed after perioperative alpha- and beta-adrenergic blockade with phenoxybenzamine and bisoprolol.

After thorough preoperative evaluation to assess the risk for surgery and anesthesia, and appropriate perioperative management including pharmacological antihypertensive treatment, dorsal instrumentation of T8-T12 and cement augmentation of T12 prior to placing the corresponding pedicle screws did not result in hypertensive crisis or hemodynamic instability due to the release of catecholamines from metastatic lesions.

To the authors' knowledge, this is the first report describing cement-augmentation in combination with dorsal instrumentation to prevent osteolytic vertebral collapse in a patient with metastatic pheochromocytoma. With appropriate preoperative measures, cement-augmented dorsal instrumentation represents a safe approach to stabilize vertebral bodies with metastatic malignant pheochromocytoma. Nevertheless, direct manipulation of metastatic lesions should be avoided as far as possible in order to minimize the risk of hemodynamic complications.

## Background

Pheochromocytomas represent neuroendocrine tumors that originate from catecholamine- producing chromaffin tissues [1]. In 80-85% of cases, pheochromocytoma is located in the adrenal gland [2]. Extra-adrenal location of pheochromocytoma accounts for 15-20%, mostly arising from intraabdominal sympathetic ganglia (therefore sometimes referred to as paraganglioma) [2]. The reported incidence of pheochromocytoma ranges from 0.2 to 0.9 per 100,000 persons annually [1,2]. Malignancy occurs in approximately 10-20% of the cases and is defined by the presence of metastases and local invasion rather than by histological criteria [3,4]. For malignant pheochromocytoma, the 5-year survival rate is reported to be up to 40% [1,5]. Malignant pheochromocytoma is known to metastasize to bone, liver, lungs, and lymph nodes. Although osseous metastases are found prevalently, involvement of the spine is considered an exceptional rarity [6-10]. In this case report, the authors present a strategy for operative stabilization of metastatic pheochromocytoma of the thoracic spine using dorsal instrumentation combined with cement augmentation of the affected segments following preoperative alpha- and beta-adrenergic receptor blockade.

## Case presentation

At the age of 48 years, the female patient presented with paroxysmal severe headaches associated with hypertensive peaks, pallor, and subsequent tremor. After laboratory testing and radiological imaging, the patient was diagnosed with extra-adrenal pheochromocytoma located retroperitoneally in the right ventro-lateral region at the level of L2. The tumor was surgically removed and the diagnosis was confirmed by histo-pathological analysis. At that time, there was no evidence for metastases neither to local lymph nodes nor to remote tissues.

The patient remained asymptomatic for four years, but then began to suffer from recurrent palpitations, paresthesia, headache, fatique, and flushing. These episodes were accompanied by increased blood pressure levels above 200/100 mmHg. Subsequent diagnostics revealed recurrence of the pheochromocytoma in form of multiple osseous metastases. The patient was admitted to the hospital due to recurrent hypertensive crises and anti-hypertensive medication was established by blockade of alpha- (phenoxybenzamine) and beta-adrenergic receptors (low-dose bisoprolol), and the calcium-channel blocker dihydropyridine. A chemotherapy with a tyrosine kinase inhibitor (sunitinib) was initiated.

**Figure 1:**
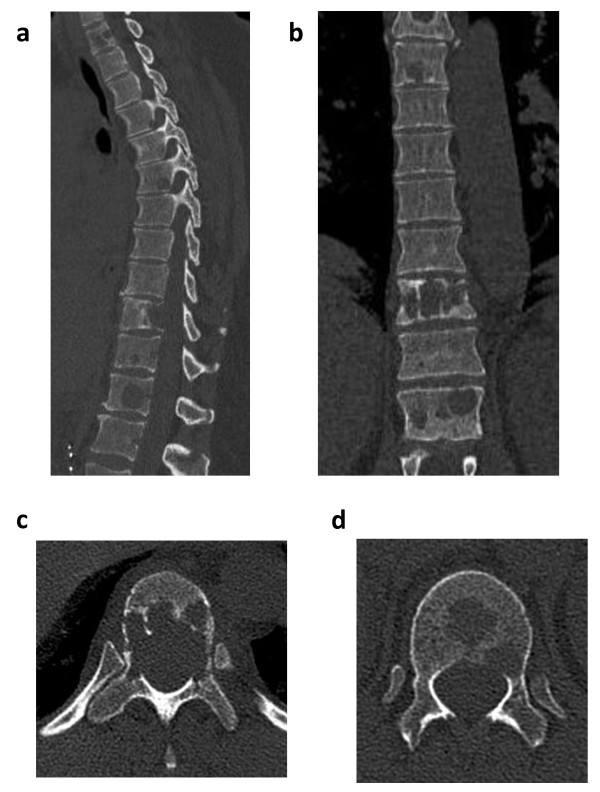
**Computed tomography (CT) of the thoracic spine**. Sagittal (**a**) and coronary (**b**) CT scans revealing multiple osteolytic lesions. **c**. Axial CT image of T10 depicting destruction of the rear edge without spinal obstruction. **d**. Axial image of the CT scan of T12 showing osteolytic lesions centrally and in proximity of the left pedicle.

^123^Metaiodobenzylguanidine scan, magnetic resonance imaging (MRI), and computed tomography (CT) scans (Figure 1) revealed multiple osteolytic lesions of the entire spine, especially prominent in T1, T5, T10, and T12. Moreover, lesions were detected in the osseous pelvis, both proximal femora, the right humerus, and the left thoracic wall. In contrast, recurrence of the primary tumor in the right retroperitoneal lumbar region was not detected. In the case of T10, CT and MRI scans showed an instable fracture of the vertebral body with entire destruction of the rear edge without spinal obstruction and a pathologic fracture of the left pedicle (Figure 1a, c). For T12, the CT scan revealed osteolytic lesions in the center of the vertebral body and another metastasis in proximity of the left pedicle (Figure 1d). Thus, the indication for operative stabilization was given to prevent the collapse of vertebral body T10 with consecutive spinal stenosis and spinal cord compression. The patient's typical symptoms consistent with pheochromocytoma in the absence of recurrence of the primary tumor indicated endocrinological activity of the metastases, implying a high risk for surgery and anesthesia. Therefore, preoperative assessment besides ECG, routine laboratory tests (electrolytes, kidney function tests, complete blood count), and chest radiograph included endocrinological evaluation (chromogranin A, metanephrines, catecholamines) and transesophageal echocardiography (TEE) for assessment of the patient's cardiac function. Furthermore, the antihypertensive treatment was adjusted, aiming for full blood pressure control with low normal values for at least one week, absence of ST-segment elevations in ECG, and less than one ventricular premature complex (VPC) over five minutes in Holter ECG monitoring.

Eventually, the patient underwent surgery for stabilization of the thoracic spine. After induction of general anesthesia and intubation, the patient was placed in a prone position for dorsal access to the lower thoracic spine. For posterior approach, the paraspinal muscles were detached subperiostally on each side after midline longitudinal incision over the spinous processes. The pedicle entry points of T8, T9, T11, and T12 were exposed by lateral dissection. At first, the pedicle screws (USS II, Synthes, Oberdorf, Switzerland) were placed bilaterally in T8 and T9, followed by pedicle screw insertion in T11. Since the patient did not react with hemodynamic fluctuations to the placement of the cephalad pedicle screws, cement-augmentation prior to pedicle screw placement to T12 was employed. Likewise, neither cement injection nor screw insertion resulted in significant alterations of blood pressure or cardiac dysfunction. Visual control with an image converter showed the optimal position of all pedicle screws so that the screws were then connected by the transfixation rods (USS II, Synthes) and the wound was closed in multiple layers technique.

**Figure 2:**
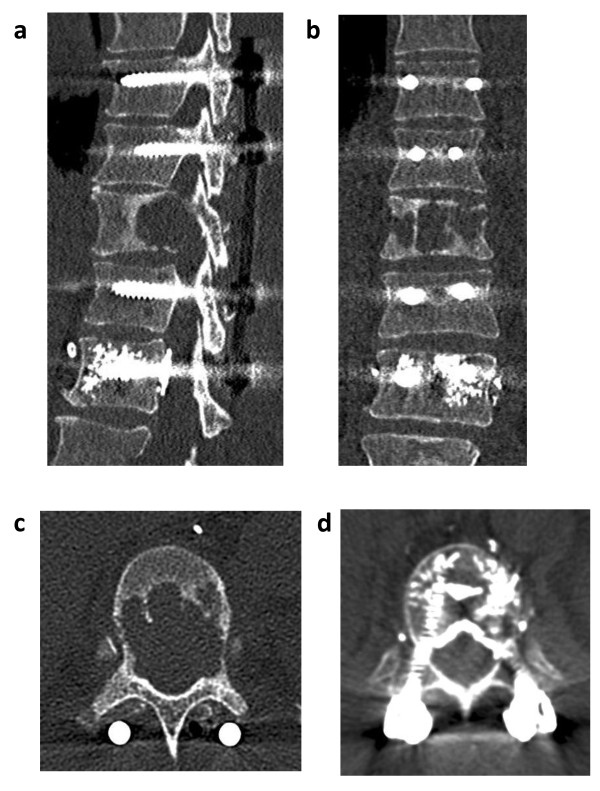
**Postoperative computed tomography (CT) of the thoracic spine**. Sagittal (**a**) and coronary (**b**) CT scans after cement-augmented dorsal instrumentation of T8-T12. (**c**) The postoperative axial imaging (CT) of T10 reveals identical results with respect to obstruction of the spinal canal, as compared to the preoperative state. (**d**) Axial CT scan of T12 showing cement augmentation prior to pedicle screw placement.

Postoperatively, the patient was referred to the ICU. The further course was uneventful and the patient was transferred to the regular ward on postoperative day one. A CT scan after mobilization of the patient confirmed the correct position of the implants and showed unaltered results for T10 with respect to vertebral body height and spinal obstruction, and no signs of cement extrusion at the segment of T12 (Figure 2). Following wound healing, palliative chemotherapy with sunitinib was restarted. In addition, the patient underwent auxiliary radiation therapy after suture removal. As the patient did not develop severe adverse effects to the initial radiation therapy cycles she was discharged and further monitored on an outpatient basis.

## Discussion

Pheochromocytoma is a neuroendocrine tumor of the medulla of the adrenal glands or extra-adrenal so-called chromaffin tissue, which synthesizes and releases catecholamines [1]. Although malignant pheochromocytoma is known to metastasize prevalently to bones, involvement of the spine is rather uncommon [6-10]. Thus, only a few reports exist describing operative approaches of stabilizing metastatic spinal pheochromocytoma and evidence-based protocols for operative strategies and the preoperative management are not available.

Since metastatic lesions from pheochromocytoma have considerable potential to be endocrinologically active in terms of producing, storing and secreting catecholamines any type of surgical manipulation bears the risk of unwanted release of catecholamines and consecutive hemodynamic complications. For surgery of primary pheochromocytoma, it has been shown that catecholamine release can be triggered by direct manipulation during dissection/extirpation of the tumor and by increase of pressure of the abdominal cavity, as it can occur by induction of a pneumoperitoneum for laparoscopy or palpation of the abdomen [11]. In addition to direct manipulation of the tumor, application of cement may increase pressure on the osseous metastatic lesion [12], and thereby cause catecholamine release. Moreover, thermal necrosis of surrounding tissue after cement application [13] might aggravate catecholamine release. Therefore, the technique of cement augmentation may bear an additional risk of unwanted release of catecholamines, as compared to simple decompression/resection and instrumented fusion. On the other hand, it is important for the stability and appropriate anchorage of pedicle screws that a mechanically sound interface is created by cement augmentation. Other factors that predispose to intraoperative hypertension besides direct manipulation of the tumor include high norepinephrine production, large tumor size (> 4 cm), a high blood pressure at presentation and after alpha-adrenergic receptor blockade (MAP > 100 mm Hg), and a pronounced postural drop (>10 mm Hg) after alpha-receptor blockade [14,15]. Interestingly, there are differences in neurochemical and secretory phenotypes of catecholamine-producing tumors. In particular, tumors predominantly containing norepinephrine are characterized by poorly differentiated secretory pathways resulting in unregulated catecholamine release, in contrast to tumors that predominantly produce epinephrine or metanephrine [16]. With respect to the risk of hemodynamic instability during surgery of patients with pheochromocytoma, there are only data available on surgical resection of the primary tumor but not on the treatment of metastatic lesions. For the pre-operative management, it has been shown that alpha-receptor blockade has a significant impact on the prevalence of perioperative complications (3% with blockade vs. 69% without alpha-adrenoceptor blockade; [17]). However, there are no randomized prospective studies that are large enough to establish the most effective drug regimen before surgery. Traditional regimens include an alpha-adrenergic receptor blockade prior to surgery with phenoxybenzamine, prazosin or phentolamine to minimize the risk of severe intraoperative hypertension [18]. Others suggest a combined alpha-/beta-adrenergic blockade to additionally prevent tachyarrhythmia [19]. In this context, it is important to note that sole beta-adrenergic blockade must not be employed since such treatment might result in severe adverse effects, such as refractory hypertension or acute myocardial necrosis due to unopposed alpha-adrenergic agonism [20,21]. Furthermore, patients with pheochromocytoma should be considered to be volume depleted because of a chronically elevated adrenergic state, which may lead to an inhibition of renin-angiotensin activity, resulting in excessive fluid loss [22]. In the present case, phenoxybenzamine was administered for non-specific, irreversible alpha-adrenergic blockade in combination with low dose selective blockade of beta1-receptors by bisoprolol.

With respect to the surgical procedure of metastatic pheochromocytoma of the spine, several procedures have been described, including laminectomy, surgical debulking, pre-operative embolization, corporectomy and reconstruction of the pathological vertebral body, anterior plating, or combined anterior and posterior stabilization of segments of the cervical spine [6,9,10,23,24]. However, to the knowledge of the authors, no reports exist describing dorsal stabilization combined with cement-augmentation for metastatic pheochromocytoma of the spine. Besides its primary indication for the treatment osteoporotic vertebral compression fractures, the use of cement in terms of percutaneous vertebroplasty has been suggested as a treatment option for spinal tumoral osteolysis. It has been demonstrated that percutaneous vertebroplasty provides immediate and long-term pain relief and restores biomechanical stability of structurally weakened vertebrae [25]. It can be combined with chemotherapy, radiation therapy, tumor ablation, and conventional surgical techniques [25]. Therefore, the use of cement for the treatment of osteolytic spinal lesions may represent a useful adjunct to the therapeutic arsenal in the palliative treatment of spinal metastases.

In the present case, cement injection into metastatic lesions and subsequent insertion of the corresponding pedicle screws did not result in significant fluctuations of blood pressure or heart rate although the metastases were considered to be hormonally active. The postoperative treatment should be adjusted to the individual patient situation, but adjuvant radiation therapy or combined radiochemotherapy should be taken into consideration as osseous lesions from pheochromocytoma tend to respond well to radiation therapy [9,26,27].

## Conclusion

Based on the anecdotal experience of the authors, cement-augmented dorsal instrumentation represents a safe approach for stabilization of metastatic pheochromocytoma of the thoracic und lumbar spine. With appropriate pretreatment (alpha- and beta-adrenergic pharmacological blockade) and preoperative diagnostic assessments the risk for hemodynamic instability can be minimized. Nevertheless, unnecessary manipulation of metastatic lesions should be omitted, not only to avoid further dissemination of neoplastic cells, but, most importantly, to prevent the unwanted release of catecholamines and subsequent adverse hemodynamic complications.

## Consent

Written informed consent was obtained from the patient for publication of this case report.

## Competing interests

The authors declare that they have no competing interests. In particular, the authors do not have any specific conflict of interest related to spine studies and the vendor of the spine system used.

## Authors' contributions

DR and CMLW wrote the manuscript. EB, LUZ, DRS, WB, CO, GAW and HPS contributed to the preparation of the manuscript. All authors read and approved the final manuscript.
